# Corn Stover Biochar Amendment Enhances Nitrogen and Phosphorus Transformations, Microbial Community Diversity, and Enzyme Activities in Agricultural Soil

**DOI:** 10.3390/plants14172787

**Published:** 2025-09-05

**Authors:** Baihui Li, Jie Zhang, Tingting Chang, Qianqian Wu, Hanyu Zheng, Dong Zhang

**Affiliations:** College of Agricultural Science and Engineering, Hohai University, Nanjing 210098, China; seraphinahui@163.com (B.L.); doris_wqq@163.com (Q.W.); hanyu_zheng@yeah.net (H.Z.); shudong1128@gmail.com (D.Z.)

**Keywords:** biochar, vertical migration, microbial community, enzyme activity

## Abstract

Corn stover biochar amendment significantly influences nitrogen (N) and phosphorus (P) transformations, microbial community composition, and enzyme activities in continuous cropping soils. This study aimed to identify the optimal biochar application rate for enhancing N and P nutrient availability in *Solanum lycopersicum* L. continuous cropping systems, providing theoretical and technical foundations for mitigating continuous cropping obstacles. A soil experiment under rain-out shelters employed four treatments: 1% biochar (BA1), 3% biochar (BA3), 5% biochar (BA5), and a non-amended control (BA0). The results indicated that biochar amendment significantly elevated available phosphorus content in the soil while effectively suppressing its vertical migration; nitrate N content increased under BA1 treatment but decreased in the BA3 and BA5 groups; and the strength of the inhibition effect of biochar treatment on the vertical migration of nitrate N was BA1 > BA5 > BA0 > BA3. The addition of biochar treatment had no significant effect on the content of ammonium N but could inhibit the vertical migration of ammonium N. The addition of biochar treatment could increase the soil’s ammonium N content. The addition of biochar treatment increased soil catalase and urease and sucrase activities, decreased alkaline phosphatase activity, led to the promotion of nitrate reductase activity at low doses and its inhibition at high doses, and resulted in BA1 treatment having the largest soil enzyme index (SEI), which was the most favorable to increase the overall level of soil enzyme activities. Biochar significantly increased the relative abundance of *Patescibacteria* and *Ciliophora* while reducing *Gemmatimonadota*, *Acidobacteriota*, *Nitrospirota*, *Ascomycota*, and *Chlorophyta*. Comprehensive evaluation using gray relational analysis (GRA) demonstrated that the addition of 5% biochar resulted in the optimal overall performance, enhancing nitrogen and phosphorus transformation, improving microbial community structure, and harmonizing enzyme activities, thereby exhibiting considerable potential for alleviating the nutrient limitations of nitrogen and phosphorus in continuous cropping soils.

## 1. Introduction

Nitrogen and phosphorus are essential macronutrients crucial for plant growth and sustaining crop productivity [1,2]. However, in intensive vegetable production systems, continuous cropping practices are widely adopted to maximize economic returns. Numerous studies indicate that prolonged monocropping severely degrades soil structure, manifesting as increased bulk density, reduced air-filled porosity, and disrupted N/P stoichiometry [3,4]. These alterations impair root nutrient acquisition and plant development, ultimately hindering the sustainable development of protected vegetable cultivation [5]. Biochar amendment offers a promising solution by enhancing soil nutrient use efficiency, suppressing pathogenic fungi proliferation [6], and facilitating N and P turnover to alleviate continuous cropping obstacles.

Corn stover biochar is thermally produced with corn stover under oxygen-limited conditions. The advantage of corn stover utilization is its wide abundance and low cost, and it therefore provides a plentiful, renewable, and cost-effective feedstock for biochar production. Corn stover biochar, with high stability and a strong aroma, has a highly organic carbon and aperture structure, a large surface area with various functional groups, and a higher cation-exchange capacity [7]. Fuertes et al. [8] reported that biochar from corn stover pyrolysis at a peak temperature of 550 °C was highly aromatic and had low H/C and O/C molar ratios For instance, the corn straw-derived biochar used in this study is particularly noted for its well-developed pore structure and significant nutrient retention capacity, making it highly suitable for soil amendment. Its application in soil remediation improves soil porosity, adsorbs root exudates, provides favorable habitats for beneficial microbes, and suppresses soil-borne pathogens [9]. However, it is important to note that the high pH characteristic of corn straw biochar may pose limitations for use in already alkaline soils.

Extensive research confirms that biochar enhances nitrogen and phosphorus availability by modifying soil properties and regulating key biochemical processes in N and P cycling [10,11,12,13]. Specifically, biochar mitigates nutrient leaching in continuous cropping systems through adsorption mechanisms that effectively inhibit vertical migration of phosphorus and nitrate [14]. Concurrently, Liang et al. [15] demonstrated that biochar amendment increases soil nitrogen availability while reducing N losses. Yang et al. [16] further established biochar’s capacity to sequester soil phosphorus, significantly elevate organic P content, and facilitate the conversion of inorganic P to labile phosphorus forms.

Research indicates that biochar amendment significantly influences soil microbial activity and community composition, consequently altering nutrient content and availability [17,18]. Diverse microbial communities accelerate organic matter decomposition, providing plant-available nitrate and ammonium nitrogen alongside microbial metabolites, thereby playing pivotal roles in nitrogen and phosphorus cycling [19]. Xu et al. [20] reported increased Bacteroidetes abundance following biochar application, Dempster et al. [21] paradoxically observed reduced soil microbial biomass. Vanek et al. [22] demonstrated that biochar enhances phosphorus bioavailability via interactions with arbuscular mycorrhizal (AM) fungi, facilitating phosphorus uptake in common beans. Similarly, Wang et al. [23] documented increased abundance of nitrogen-cycling functional microbiota in degraded agricultural soils amended with rice straw and sludge-derived biochars. Critically, biochar’s ameliorative effects exhibit dose dependency [24], as excessive application may potentially compromise nutrient bioavailability or disrupt microbial functionality [25].

Soil microbial-driven nitrogen and phosphorus transformations are primarily mediated by metabolic enzymes within microorganisms. Extensive research documents that biochar amendment significantly influences the activity of key enzymes involved in N and P cycling [26,27,28]. Notably, biochar frequently enhances phosphatase activity [19]—which can catalyze the hydrolysis of organically bound phosphorus, thereby potentially increasing soil P bioavailability. Supporting this, Khadem and Raiesi [29] demonstrated that maize-derived biochar stimulated alkaline phosphatase activity, accelerating organic phosphate mineralization in arid soils. Conversely, Liu et al. [30] observed suppressed activities of certain N-cycling enzymes following biochar application. Pokharel et al. [31] reported divergent findings, indicating that biochar enhanced urease activities, while concurrently inhibiting alkaline phosphatase synthesis.

Current research mostly focuses on the effects of biochar dosage on specific soil indicators but lacks a systematic quantification of its comprehensive effects. This study targeted yellow-brown soil that had been cultivated for three consecutive tomato crops. Four different biochar application rates were established to examine variations in soil microbial community composition, key enzyme activities, and nitrogen and phosphorus contents. A comprehensive evaluation model was developed using gray relational analysis (GRA) to identify (1) the vertical migration pattern of N and P and the driving factors of N and P content in continuous cropping soil under biochar addition and (2) the optimal dosage of nitrogen and phosphorus nutrients in continuous cropping soil improved by biochar. It provides a reference for the improvement of continuous crop soil and the popularization and application of biochar.

## 2. Results

### 2.1. Effect of Biochar Amendment on Vertical Transport of Soil-Available P, Nitrate N, and Ammonium N

**Figure 1:**
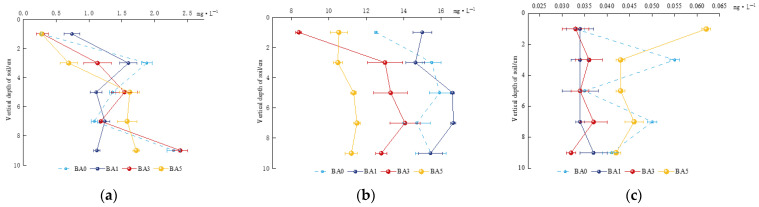
Vertical migration changes in soil-available phosphorus, nitrate N, and ammonium N. (**a**) Available phosphorus; (**b**) nitrate N; (**c**) ammonium N.

As shown in Figure 1a,b, the increase in available phosphorus and nitrate N in 8–10 cm compared to those in the 0–2 cm soil layer under BA0, BA1, BA3, and BA5 treatments were 990.48%, 51.35%, 724.14%, and 514.29% and 23.40%, 2.94%, 52.56%, and 6.36%. BA0 treatment indicated that available phosphorus and nitrate N in natural soil were easily transported downward and enriched in the deep layer. The addition of biochar could effectively inhibit the vertical migration of available phosphorus, and the effect size was BA1 > BA5 > BA3 > BA0. Conversely, biochar differentially impacted the vertical migration of nitrate N; BA1 and BA5 treatments inhibited the vertical migration of nitrate N, whereas BA3 promoted the vertical migration of nitrate N, and the effect was 2.2 times more than that of BA0. The BA5 treatment had the highest content of ammonium N at a depth of 0–2 cm, and its content was twice that of BA0. Biochar addition could inhibit the vertical migration of ammonium N, and the content of ammonium N was similar to that of BA0 at a depth of 8–10 cm, indicating that the BA5 treatment could effectively inhibit the vertical migration of ammonium N. The changes in the content of ammonium N at all depths of the soil in the BA1 and BA3 treatments were smaller than those of BA0 (Figure 1c), which indicated that the BA1 and BA3 treatments also inhibited the vertical migration of ammonium N. The BA5 treatment had the highest content at a depth of 0–2 cm, which was two times as effective as BA0.

### 2.2. Effect of Biochar Amendment on Soil-Available P, Nitrate N, and Ammonium N Content

Biochar treatments significantly elevated soil-available phosphorus content (Table 1); BA1 treatment increased the content of nitrate N, but there was no significant difference from BA0 treatment; BA3 treatment reduced the content of nitrate N, which decreased by 26.64%; BA5 treatment significantly reduced the content of nitrate N by 33.99%; and biochar did not significantly affect ammonium N.

### 2.3. Effects of Biochar Amendment on Soil Field Capacity

**Figure 2:**
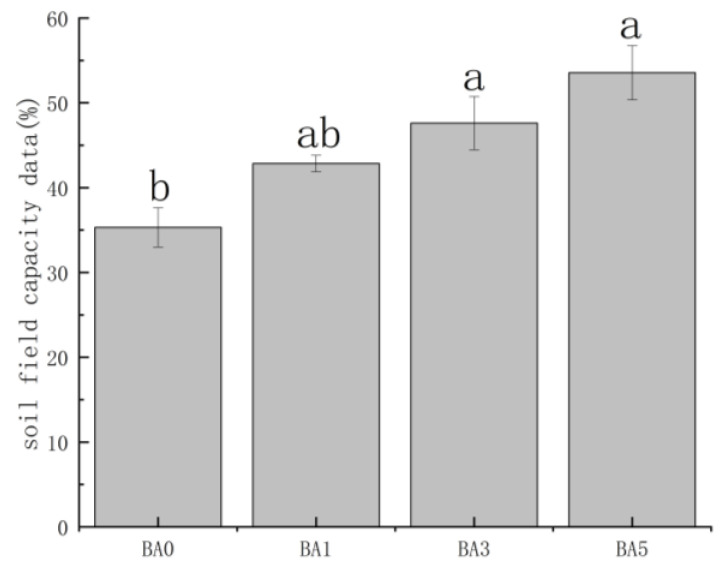
Soil field capacity as influenced by different biochar amendment rates. Values are means. Different letters indicate significant differences (*p* < 0.05).

The application of biochar significantly influenced the soil’s water retention capacity, as measured by the field capacity (FC). As illustrated in Figure 2, the field capacity exhibited a pronounced increasing trend with higher biochar application rates. The FC of the control treatment (BA0) was measured at 35.3%. The addition of 1% (BA1), 3% (BA3), and 5% (BA5) biochar progressively increased the field capacity to 42.8%, 47.9%, and 53.5%, respectively. Statistical analysis confirmed that all biochar-amended treatments resulted in a significantly higher field capacity (*p* < 0.05) compared to the control, with the BA5 treatment demonstrating the most substantial enhancement.

### 2.4. Effects of Biochar Amendment on Microbial Community Composition

#### 2.4.1. Effect of Biochar Amendment on the Relative Abundance Composition of Microorganisms

**Figure 3:**
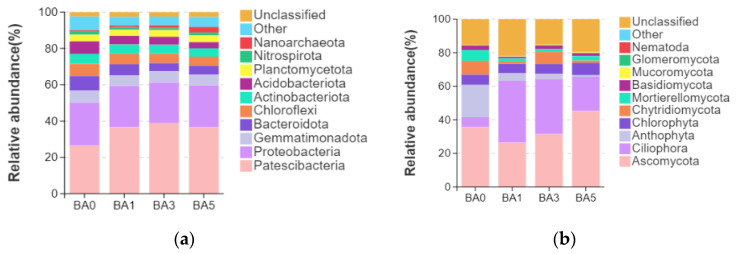
Effect of biochar on bacterial and fungal phyla in soil: (**a**) bacterial; (**b**) fungi.

The composition of the bacterial and fungal phyla under the application of different biochar treatments to the continuous soil is analyzed in Figure 3. Under the bacterial phyla, the application of biochar treatments increased the relative abundance of the *Patescibacteria* phyla compared to BA0; the application decreased the relative abundance of *Gemmatimonadota*, *Bacteroidota*, *Acidobateriota*, *Nitrospirota*, and *Chloroflexi* phyla. For fungal phylum classification, biochar application decreased the relative abundance of *Ascomycota* phylum compared to BA0; BA1 treatment significantly increased the relative abundance of *Ciliophora* phylum, and BA3 treatment increased the relative abundance of *Ciliophora* phylum.

#### 2.4.2. Effect of Biochar Amendment on Dominant Microbial Populations

**Figure 4:**
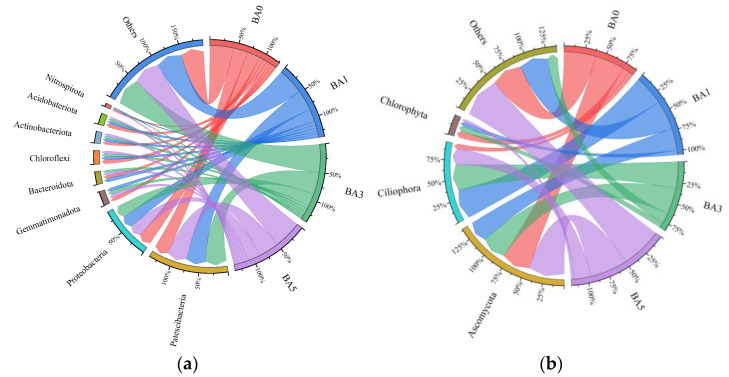
String diagram of phylum bacteria and phylum fungi: (**a**) bacteria; (**b**) fungi.

As can be seen in Figure 4, the major bacterial phyla of the soil bacterial phyla measured were *Patescibacteria*, *Proteobacteria*, *Gemmatimonadota*, *Bacteroidetes*, *Chloroflexi*, *Actinobacteria*, *Acidobateriota*, and *Nitrospirota* phylum, and the cumulative abundance of the BA0, BA1, BA3, and BA5 treatments amounted to 84.23%, 88.89%, 87.90%, and 85.81%, respectively. The top three dominant species groups in each treatment of fungi were *Ascomycota*, *Ciliophora*, and *Chlorophyta*, and the cumulative abundance of the top three phyla in the BA0, BA1, BA3, and BA5 treatments amounted to 60.73%, 64.11%, 87.70%, and 49.35%, respectively.

**Figure 5:**
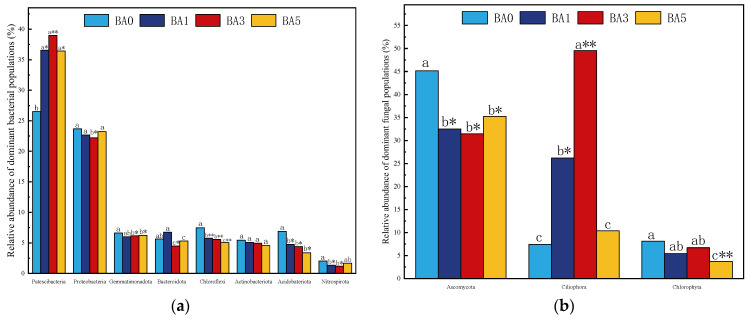
Dominant soil microbiome abundance (%): (**a**) bacteria; (**b**) fungi. Note: The lowercase letters indicate the significance analysis results of four treatments in the greenhouse (*p* < 0.05); Where mark “*” indicates a significant difference (*p* < 0.05) and “**” indicates a highly significant difference (*p* < 0.01) compared to the BA0 treatment.

Biochar application had significant effects on the dominant fungal and bacterial phyla compared to no biochar application (Figure 5). For bacterial phyla, biochar application significantly or very significantly increased the relative abundance of *Patescibacteria* phylum, significantly decreased the relative abundance of *Gemmatimonadota*, *Acidobateriota*, and *Nitrospirota* phyla, and very significantly decreased the relative abundance of *Chloroflexi* phyla compared to BA0. The BA3 treatment significantly reduced the relative abundance of the *Proteobacteria* phylum, and there were no significant differences between BA1, BA5, and the unapplied biochar treatment.

On the fungal phylum, the application of biochar significantly reduced the abundance of *Ascomycota* phylum, and the abundance of *Ascomycota* phylum in the BA1, BA3, and BA5 treatments decreased by 28.36%, 28.36%, and 22.37%, respectively, compared with the BA0 treatment. The treatments of BA1 and BA3 significantly increased the abundance of the *Ciliophora* phylum, and the abundance of the *Ciliophora* phylum was significantly increased by the application of 3% biochar treatment. There was no significant difference between the BA5 and BA0 treatments. The highest abundance was observed in the BA5 and BA0 treatments, with no significant difference between them. The BA5 treatment decreased the relative abundance of *Chlorophyta* very significantly.

### 2.5. Effect of Biochar Amendment on Enzyme Activity

As shown in Table 2, there were differences in the effects of different biochar addition treatments on soil enzyme activities. Compared with BA0, the activities of catalase and sucrase in soil increased by 2.06% to 7.28% and 26.16% to 58.65%, respectively, while alkaline phosphatase activity was significantly decreased by 23.68% to 34.21%, and this trend in increase or decrease increased with the increase in addition. It is noteworthy that nitrate reductase activity increased by 26.67% to 31.11% in BA1 and BA3 treatments, while it decreased by 40.00% in BA5 treatment. The activities of catalase and urease were significantly or highly significantly increased under BA5 treatment compared to BA0, but the effects of BA1 and BA3 treatments were not significant. Both the sucrase activity and alkaline phosphatase activity of soil treated with added biochar were significantly different from the BA0 group. Nitrate reductase activity showed greater fluctuation with the increase in biochar addition compared to BA0.

The soil enzyme index (SEI) reflects the combined value of soil enzyme activities after the application of different biochar additions. As shown in Table 3, the weighting coefficients of soil catalase, urease, sucrase, and neutral phosphatase were 0.30, 0.32, 0.31, 0.18, and 0.21, respectively. As shown in Table 4, the SEI exhibited an initial increase followed by a decline with rising biochar addition, and the SEI was BA1 > BA5 > BA3 > BA0. The SEI reached the maximum value when biochar was 1%, which was most favorable to improve the overall level of soil enzyme activity.

### 2.6. Correlation Analysis

**Figure 6:**
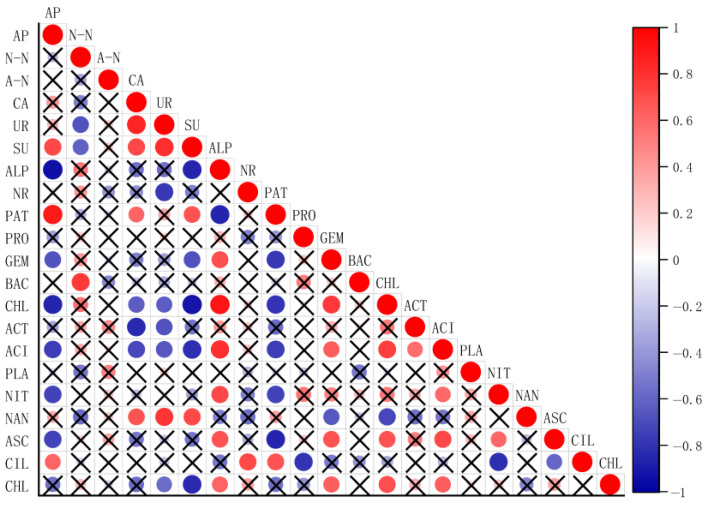
Heat map for correlation analysis. Note: *p* < 0.01 means the two are correlated, and the x in the figure means *p* > 0.01, not correlated. AP stands for available phosphorus; N-N stands for soil nitrate N; A-N stands for soil ammonium N; CA stands for catalase enzyme; UR stands for urease enzyme; SU stands for sucrase; ALP stands for alkaline phosphatase; NR stands for nitrate reductase; PAT stands for *Patescibacteria*; PRO stands for *Proteobacteria*; GEM stands for *Gemmatimonadota*; BAC stands for *Bacteroidota*; CHL stands for *Chloroflexi*; ACT stands for *Actinobacteriota*; ACI stands for *Acidobateriota*; PLA stands for *Planctomycetota*; NIT stands for *Nitrospirota*; NAN stands for *Nanoarchaeota*; ASC stands for *Ascomycota*; CIL stands for *Ciliophora*; and CHL stands for *Chlorophyta*.

From the correlation analysis (Figure 6), soil-available phosphorus was negatively correlated with alkaline phosphatase, *Gemmatimonadota*, *Chloroflexi*, *Acidobateriota*, *Nitrospirota*, and *Ascomycota* phylum, and with positively correlated sucrase, *Patescibacteria*, *Ciliophora* phylum; soil nitrate N correlated negatively with urease and sucrase activities but positively with *Bacteroidota* abundance.

### 2.7. Gray Correlation Analysis

Due to the differences in soil nitrogen and phosphorus content, microbial dominant population abundance, and enzyme activity among treatments was evaluated in order to better evaluate the effect of biochar addition, the average concentration of soil-available phosphorus, nitrate N, ammoniacal N, nitrogen and phosphorus cycling-related microbial phylum and soil enzyme activity indicators, with a total of 14 indicators from four treatments used to construct a gray system (Table 5). Soil improvement efficacy positively correlated with total weighted correlation magnitude. Experimental treatments were ranked by gray correlation degree as BA5 > BA1 > BA3 > BA0 (Table 6). The results showed that the addition of 5% biochar had the best comprehensive performance effect in this experiment.

## 3. Discussion

### 3.1. Effects of Biochar Amendment on Vertical Migration and Concentrations of Soil-Available Phosphorus, Nitrate N, and Ammoniacal N

Biochar amendment effectively enhanced soil nutrient retention while suppressing nitrogen and phosphorus leaching. The increased soil available phosphorus content following biochar application supports crop health and growth, thereby improving agricultural ecosystem productivity. This aligns with the findings of Chintala et al. [32], who demonstrated that corn stover biochar can enhance the adsorption and sequestration of available phosphorus. The pronounced ability of biochar to elevate soil-available phosphorus and reduce phosphorus loss, as observed in our study, can be attributed to its inherent properties [33]. Our experiment further demonstrated that the addition of biochar not only significantly increased the available phosphorus content but also effectively inhibited its vertical migration. This suppression may be attributed to phosphorus immobilization through adsorption or passivation mechanisms specific to biochar [34,35,36]. The alkaline nature of corn stover biochar likely plays a key role by promoting phosphate precipitation via soil pH modulation, thereby reducing leaching risks [37]. Furthermore, its microporous structure enhances the adsorption of mobile phosphorus fractions, consequently limiting downward translocation through the soil profile [38]. Although biochar amendment increased soil field capacity (Figure 2), the inhibition of phosphorus leaching is likely dominated by chemical and physical adsorption rather than by alterations in soil water dynamics.

The response of soil inorganic nitrogen to biochar amendment displayed a complex, non-linear relationship with the application rate. BA1 treatment increased soil nitrate-N content, whereas both BA3 and BA5 treatments decreased it. Concurrently, BA1 and BA5 effectively inhibited the vertical migration of nitrate-N, while BA3 promoted its downward translocation. This differential behavior may be attributed to distinct mechanisms governed by the application rate. (1) BA1 (1%): The reduction in nitrate-N leaching at this low rate is likely achieved through direct electrostatic adsorption by biochar functional groups, which effectively immobilizes nitrate ions without drastically altering soil physical properties. (2) BA3 (3%): This treatment significantly increased soil field capacity (Figure 2). We hypothesize that this enhancement in soil water retention potentially facilitates the infiltration and downward translocation of soluble nitrate, explaining the promoted vertical migration observed in our results. (3) BA5 (5%): Despite resulting in the highest soil field capacity (Figure 2), this treatment effectively inhibited nitrate leaching. This suggests that at high application rates, the mechanism shifts. The immense surface area and microporous structure of the large amount of biochar likely dominate the process, providing ample sites for the physical retention and adsorption of nitrate. Furthermore, the excessive biochar may create a complex porous network that increases the tortuosity of water flow paths, prolonging hydraulic residence time and potentially enhancing denitrification opportunities, thereby retarding vertical movement despite the high water-holding capacity. Biochar minimally affected ammonium N. Still, biochar addition could inhibit the vertical transport of ammoniacal N, probably because although ammoniacal N only accounts for a very small portion of soil nitrogen, biochar’s extensive surface area and porous structure offer ample adsorption sites for ammoniacal N [39] so that it is not easy to be leached out to downward seepage.

### 3.2. Effects of Different Biochar Amendment on Microbial Community Structure

Corn stover biochar application increased the abundance of the soil microbial community. It changed the community composition of soil bacteria and fungi to some extent [40], which is consistent with the results of this study. With the increase in biochar addition, the relative abundance of microbial community showed a tendency of increasing and then decreasing, which was due to the fact that the increase in biochar addition would promote the growth of some types of bacteria while inhibiting the growth of some bacteria, resulting in the change in soil bacterial community structure [41]. In this study, it was found that different biochar additions did not change the main composition of bacteria and fungi on the phylum. Still, corn stover biochar additions significantly increased the relative abundance of *Patescibacteria* phylum. It was suggested that the unique structure of biochar can provide a favorable place for soil bacteria to reproduce, rich in nutrients, which is conducive to the growth of the bacteria and their increase in relative abundance [42]. Meanwhile, this study found that the relative abundance of *Gemmatimonadota*, *Acidobateriota*, *Nitrospirota*, and *Chloroflexi* phylum was significantly reduced by the application of corn stover biochar, which may be because the application of biochar into the soil can improve the microecological environment of the soil to a certain extent, but it may be beneficial to the increase in the relative abundance of individual taxa only [43,44,45]. In addition, the addition of biochar treatment significantly decreased the relative abundance of the phylum *Ascomycota*. It significantly increased the relative abundance of the *Ciliophora* phylum compared to the BA0 treatment, which may be due to the fact that the bacterial proliferation facilitated by biochar may further inhibit ascomycetes through nutrient competition or antibiotic secretion, and, on the contrary, the ciliates gained their energy through ingesting the bacteria; the expansion of their populations may be directly related to the bacterial biomass increase. For example, it was found that fast-growing ciliates could cause rapid death or cyst formation of 12 pathogenic fungi, suggesting that biochar may ameliorate the barriers to succession by inhibiting the growth of pathogenic fungi, reflecting the alteration of fungal–bacterial interactions mediated by biochar [25], which is in line with the mechanism of Kolton et al. [46] on the enhancement of plant disease resistance by biochar.

### 3.3. Effect of Different Biochar Amendment on Enzyme Activity

Due to its unique physicochemical properties, corn stover biochar can alter soil physicochemical properties after application and impact soil enzyme activities to some extent. Table 3 shows that BA5 treatment significantly increased the activities of catalase and urease in continuous cropping soil in tomato compared with BA0 treatment. Additionally, the activities of catalase and urease were increased under BA1 and BA3 treatments, but the differences were not significant. Different biochar addition treatments significantly increased sucrase activity, which may be due to the promotion of microbial metabolism and organic matter decomposition through the improvement of soil aeration and organic matter stabilization [47], thus promoting the increase in soil enzyme activity. However, alkaline phosphatase activity decreased with increasing corn stover biochar addition, probably since biochar contains fewer easily decomposable components, which reduces substrate effectiveness and thus inhibits enzyme synthesis. Nitrate reductase activity increased and then decreased with the addition of corn stover biochar, which indicated that the nitrate reductase activity could be significantly increased by adding the appropriate proportion of biochar. The addition of too much biochar was detrimental to the soil nitrate reductase activity, which might be related to the fact that the excessive addition of biochar altered the pH or released inhibitory substances, thus interfering with the nitrification–denitrification equilibrium.

### 3.4. Correlation of Soil Nitrogen and Phosphorus Content with Soil Enzyme Activity and Microbial Community After Biochar Application

Soil microorganisms play a crucial role in N and P turnover [19,48], which directly impacts soil nutrient content and effectiveness [49]. The soil microbe-driven nitrogen and phosphorus transformation process is primarily driven by a series of metabolism-related enzymes (proteins) present in microorganisms. Numerous studies have found that the activities of enzymes related to both nitrogen and phosphorus cycling in the soil were significantly affected by the application of biochar [26,27,28,50]. As shown in Figure 6, urease was negatively correlated with nitrate nitrogen, which is probably because soil urease catalyzes urea decomposition and is involved in regulating biological nitrogen metabolism [51]. Additionally, alkaline phosphatase showed a negative correlation with available phosphorus, which is probably because alkaline phosphatase can catalyze the mineralization of organophosphorus compounds (e.g., phytic acid) and is involved in regulating the biological phosphorus cycle [52]. The results of this study showed that sucrase was positively correlated with available phosphorus and negatively correlated with nitrate N, and the positive correlation with available phosphorus was stronger. These relationships can be further explained by the varying influences of corn stover biochar amendment levels on microbial community structure and function. Specifically, with increasing corn stover biochar addition, the increased abundance of *Patescibacteria* may enhance sucrose metabolism and organic acid secretion, facilitating phosphorus mobilization. In contrast, the suppression of nitrifying bacteria such as *Nitrospirota* under biochar amendment likely contributed to reduced nitrate nitrogen levels, aligning with the negative correlation with sucrase activity. Feng et al. demonstrated that the addition of biochar significantly enhanced soil enzyme activity and soil nutrient levels, and that these changes in soil nutrients and physicochemical properties influenced the inter-root soil bacterial community. The effect of high additions of biochar was greater than that of low additions of biochar [53], which was similar to the results of this study.

## 4. Materials and Methods

### 4.1. Experimental Materials

The biochar used in this study was produced from corn stalks through slow pyrolysis at 500 °C under oxygen-limited conditions for 2–3 h, provided by Henan Lize Environmental Protection Technology Co., Ltd. (Henan, China). The material was subsequently crushed and sieved through a 70-mesh sieve (0.2 mm particle size) prior to use. Its key properties were as follows: pH 9.30; organic carbon 410.96 g·kg^−1^; total N 8.34 g·kg^−1^; total P 2.34 g·kg^−1^; total K 15.91 g·kg^−1^; P_2_O_5_ 5.34 g·kg^−1^; and K_2_O 19.17 g·kg^−1^.

### 4.2. Experimental Site Description

The experiments were conducted in plastic greenhouses located at the Water-saving Park, Jiangning Campus, Hohai University (31°91′ N, 118°79′ E), which lies within a north subtropical humid monsoon climate zone. The region has a mean annual temperature of 15.7 °C, with annual precipitation averaging 1072.9 mm and evaporation of 900 mm. The average annual sunshine duration is 2200 h, and the mean annual relative humidity reaches 81%. There are approximately 117.8 rainy days per year (daily rainfall ≥ 0.1 mm), and the maximum daily precipitation recorded is 299.0 mm.

### 4.3. Experimental Design and Treatments Application

**Figure 7:**
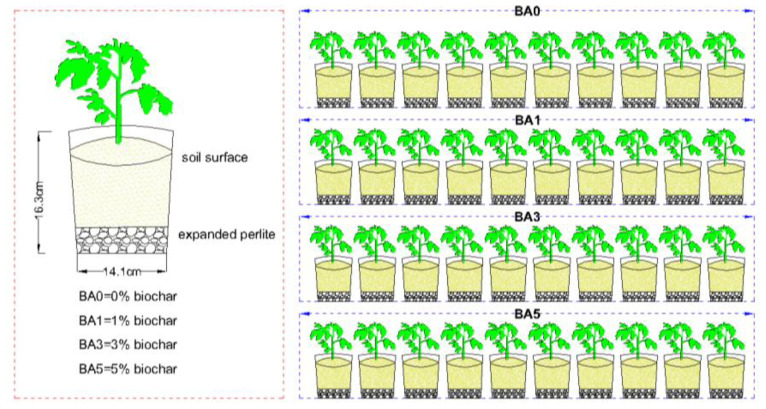
Experimental setup diagram.

Conducted from March to July 2020, the experiment included four treatments:

BA1 (1% biochar), BA3 (3% biochar), BA5 (5% biochar), and BA0 (biochar-free control), each with 10 replicates (Figure 7).

Soil was air-dried and sieved (≤2 mm) prior to amendment incorporation. Homogenized soil–amendment mixtures were loaded into 16.3 cm high × 14.1 cm diameter buckets (12 kg soil/barrel) over a 2 cm perlite base layer.

‘Cooperative 903’ tomato (45,000 plants·hm^−2^) was transplanted at the late seedling stage (one plant/bucket). Basal fertilization used 20 g of compound fertilizer (15:15:15 N:P:K) per barrel. Four fruits per inflorescence were retained, with thinning at the second-inflorescence pink stage. Routine field management was maintained.

### 4.4. Determination of Tomato Growth Indicators

Soil sampling was conducted after 105 days with three random replicates per treatment. Depth-specific fractions (0–2, 2–4, 4–6, 6–8, 8–10 cm) were collected, air-dried, sieved, and analyzed for physicochemical properties. The phosphomolybdenum blue color development method and an ultraviolet spectrophotometer were used to determine the content of soil effective phosphorus, and the content of nitrate nitrogen and ammoniacal nitrogen were determined by microtiter plate spectrophotometry.

The soil was collected from a depth of 0 to 10 cm for the analysis of microbial capacity and enzyme activities. Microbial sequences were amplified by PCR and analyzed by sequencing using microbial diversity amplicon sequencing. Total soil DNA was extracted using HiPure Soil DNA kit (model D3142, Guangzhou Magen Biotechnology Co., Ltd. (Guangzhou, China)). After genomic DNA was extracted from bacterial and fungal samples, the V3-V4 region of 16S rDNA (bacterial) and the ITS2 region of ITS (fungal) were amplified with specific primers with barcode, respectively, and the primer sequences are shown in Table 7.

Catalase activity was quantified via potassium permanganate titration; urease activity via sodium phenol-sodium hypochlorite colorimetry; sucrase activity via 3,5-dinitrosalicylic acid colorimetry; alkaline phosphatase activity via disodium phosphate colorimetry; and nitrate reductase activity via sulfanilamide colorimetry.

In order to eliminate the influence of the evaluation index scale on the factor loading of soil enzyme activity, this study used the soil enzyme index (*SEI*) to numerically synthesize and evaluate the comprehensive value of soil enzyme activity.

The formula for calculating *SEI* is as follows:

(1) $$SEI=\sum_{i=1}^{n}\omega_{i}\times SEI(x_{i}\;)$$

(2) $$\omega_{i}=\frac{c_{i}}{c}$$

where $\omega_{i}$ denotes the weight of soil enzyme activity (i); ${SEI(x}_{i})$ denotes the value of soil enzyme affiliation; $c_{i}$ represents the mean correlation coefficient between soil enzyme activity (i) and all other soil enzyme activities; and c is the sum of the average of correlation coefficients between all soil enzyme activities.

Whether the distribution of the affiliation function was ascending or descending was determined from the inhibition or promotion of soil enzyme activities by biochar. In this study, the descending distribution function was used for soil alkaline phosphatase, the ascending distribution function was used for soil catalase, urease, and sucrase, the ascending distribution function was used for nitrate reductase biochar additions of 1% and 3% (BA1, BA3) treatments, and the descending distribution function was used for 5% biochar treatment (BA5).

$SEI(x_{i})$ is calculated as follows:

(3) $$Ascending\;distribution\;function\;SEI\left(x_{i}\right)=\frac{x_{i}-x_{i\;min}}{x_{i\;max}-x_{i\;min}}$$

(4) $$degenerate\;distribution\;function\;SEI\left(x_{i}\right)=\frac{x_{i\;max}-x_{i}}{x_{i\;max}-x_{i\;min}}$$

where $x_{i}$ denotes the soil enzyme activity; $x_{i\;max}$ and $x_{i\;min}$ denote the maximum and minimum values of soil enzyme activity (i) in the treatment, respectively.

### 4.5. Gray Relational Analysis Method

Gray correlation analysis quantifies inter-curve correlations by evaluating geometric shape similarity across sequences. This method measures time-series relationships through quantitative trend dynamics analysis, calculating gray correlation degrees between a reference series and comparison series. Lower correlation occurs when factor changes diverge substantially, while convergent changes yield higher correlations. Soil improvement outcomes were comprehensively evaluated using this method.

The following is the specific calculation method:

Index weight calculation (entropy weight method):

(5) $$\omega_{j}=\frac{1-e_{j}}{\sum_{j=1}^{m}\left(1-e_{j}\right)}$$

where $e_{j}$ is the entropy value of the *J* th item.

Gray relational degree analysis:

(6) $${x\prime }_{ij}=\frac{x_{ij}}{x_{0j}}$$

(7) $${x\prime }_{ij}=1-\frac{x_{ij}}{x_{0j}}$$

(8) $$\xi_{i}\left(k\right)==\frac{min\left(i\right)min\left(k\right)\left|X_{0}\left(k\right)-X_{i}\left(k\right)\right|+\rho max\left(i\right)max\left(k\right)\left|X_{0}\left(k\right)-X_{i}\left(k\right)\right|}{\left|X_{0}\left(k\right)-X_{i}\left(k\right)\right|+\rho max\left(i\right)max\left(k\right)\left|X_{0}\left(k\right)-X_{i}\left(k\right)\right|}$$

(9) $$\gamma_{i}=\sum_{k=1}^{n}W\left(k\right)\xi_{i}(k)$$

where *k* represents the index identifier, *i* denotes the data sequence position, and ρ is the resolution coefficient set to 0.5. Gray correlation theory designates the reference series as the optimal standard for soil quality assessment. Therefore, increased correlation between evaluated indices and this reference series directly corresponds to improved soil quality status.

### 4.6. Statistical Analysis

Data processing employed Microsoft Excel 2021 and IBM SPSS Statistics 27.0, with graphical visualization using Origin 2021. Statistical analyses included univariate ANOVA with Duncan’s post hoc test (*p* < 0.05) and Pearson correlation analysis.

## 5. Conclusions

Biochar amendment effectively improved soil quality in the continuous cropping system, in a highly application-rate-dependent manner. The most integrated improvement was BA5, which significantly enhanced available phosphorus content and inhibited its vertical migration, while also reducing nitrate N content and limiting its vertical migration. In contrast, BA1 increased nitrate N but suppressed its vertical migration, whereas BA3 reduced nitrate N yet promoted its downward movement. All biochar treatments inhibited the vertical migration of ammoniacal N, though its content remained largely unaffected. Importantly, BA1 showed the highest soil enzyme activity index (SEI), indicating enhanced biochemical functionality. Biochar application also modified microbial community composition, and correlation analyses confirmed that soil microorganisms and enzymes played crucial roles in mediating nitrogen and phosphorus transformation. To facilitate practical application, it is recommended that biochar be applied as a basal amendment at a rate of 5% and thoroughly incorporated into the top 15–20 cm soil layer before planting. Attention should be paid to soil pH monitoring before large-scale application, as excessive biochar may elevate pH in alkaline-sensitive soils.

Nevertheless, this study was conducted solely on yellow-brown soil under tomato monoculture, and the physicochemical properties of the biochar were not characterized, limiting mechanistic insight into structure–function relationships. Future research should therefore involve multi-site long-term trials across different soil types and cropping systems and employ detailed biochar characterization to elucidate the mechanisms underlying biochar-induced improvements in soil nutrient cycling and microbial ecology.

## Figures and Tables

**Table 1:** Effect of different treatments on the mean concentrations of available phosphorus, nitrate N, and ammonium N in soil.

Treatment	Available Phosphorus (mg·kg^−1^)	Nitrate N (mg·kg^−1^)	Ammonium N (mg·kg^−1^)
BA0	1.2 ± 0.0060 b	15 ± 1.2 a	0.042 ± 0.0050 a
BA1	1.4 ± 0.043 a	16 ± 0.11 a	0.035 ± 0.0010 a
BA3	1.4 ± 0.021 a	12 ± 0.15 b	0.042 ± 0.011 a
BA5	1.4 ± 0.034 a	11 ± 0.13 b	0.046 ± 0.0040 a
BA0	1.2 ± 0.0060 b	15 ± 1.2 a	0.042 ± 0.0050 a

Note: values represent mean ± Standard Deviation. The lowercase letters indicate the significance analysis results of four treatments in the greenhouse; different letters in the same column represent significant differences (*p* < 0.05). BA0, BA1, BA3, and BA5 stand for no biochar, 1% biochar, 3% biochar, and 5% biochar.

**Table 2:** Comparison of different enzyme activities under different biochar addition treatments (mg·g^−1^·24 h^−1^).

Treatment	Catalase Enzyme	Urease Enzyme	Sucrase	Alkaline Phosphatase	Nitrate Reductase
BA0	6.32 ± 0.08 b	0.24 ± 0.02 b	43.00 ± 0.63 c	0.38 ± 0.01 a	0.45 ± 0.02 b
BA1	6.45 ± 0.07 ab	0.24 ± 0.01 b	54.25 ± 2.12 b **	0.29 ± 0.02 b **	0.57 ± 0.03 a
BA3	6.51 ± 0.29 ab	0.27 ± 0.01 b	56.55 ± 1.00 b **	0.26 ± 0.02 c **	0.59 ± 0.07 a *
BA5	6.78 ± 0.14 a *	0.35 ± 0.02 a **	68.22 ± 1.20 a **	0.25 ± 0.02 c **	0.27 ± 0.04 c **

Note: values represent mean ± Standard Deviation. The lowercase letters indicate the significance analysis results of four treatments in the greenhouse; different letters in the same column represent significant differences (*p* < 0.05). BA0, BA1, BA3, and BA5 stand for no biochar, 1% biochar, 3% biochar, and 5% biochar, where mark “*” indicates a significant difference (*p* < 0.05) and “**” indicates a highly significant difference (*p* < 0.01) compared to the BA0 treatment.

**Table 3:** Correlation coefficients and weighting coefficients of soil enzyme activities under different biochar addition treatments.

Indicators	Catalase Enzyme	Urease Enzyme	Sucrase	Alkaline Phosphatase	Nitrate Reductase
catalase enzyme	1.00				
urease enzyme	0.83	1.00			
sucrase	0.69	0.82	1.00		
alkaline phosphatase	0.49	0.55	0.86	1.00	
nitrate reductase	0.46	0.78	0.49	0.08	1.00
mean value of correlation coefficient	0.62	0.59	0.72	0.50	0.45
weighting factor	0.28	0.27	0.32	0.22	0.20

**Table 4:** Affiliation values of soil enzyme activities and soil enzyme index under different biochar addition treatments.

Treatment	SEI Affiliation Value					Soil Enzyme Index (SEI)	Soil Enzyme Index Ranking
	Catalase Enzyme	Urease Enzyme	Sucrase	Alkaline Phosphatase	Nitrate Reductase		
BA0	0.35	0.39	0.37	0.38	0.41	0.49	4
BA1	0.56	0.39	0.50	0.30	0.65	0.62	1
BA3	0.37	0.42	0.58	0.22	0.39	0.53	3
BA5	0.38	0.29	0.59	0.43	0.68	0.61	2

**Table 5:** Indicator weighting data.

Soil Index	Ej	Weight
AP	0.9022	0.0739
N-N	0.8594	0.1062
A-N	0.8604	0.1054
Patescibacteria	0.9024	0.0737
Gemmatimonadota	0.9294	0.0533
Bacteroidota	0.9124	0.0662
Chloroflexi	0.9243	0.0572
Acidobateriota	0.9539	0.0348
Nitrospirota	0.9506	0.0373
Ascomycota	0.9383	0.0466
Ciliophora	0.8153	0.1395
Urease enzyme	0.9370	0.0476
Sucrase	0.8802	0.0904
Alkaline phosphatase	0.9099	0.0680

**Table 6:** Weighted correlation coefficients of soil indicators.

Treatment	WGCD	WO
BA0	0.8123	4
BA1	0.8666	2
BA3	0.8638	3
BA5	0.8747	1

**Table 7:** Amplification primers and reaction conditions of PCR.

Types	Sequencing Region	Name of Primer	Primer Sequence (5′-3′)
16S bacterium	V3-V4	341F	CCTACGGGNGGCWGCAG
		806R	GGACTACHVGGGTATCTAAT
ITS Fungal	ITS2	ITS3_KYO2	GATGAAGAACGYAGYRAA
		ITS4	TCCTCCGCTTATTGATATGC

## Data Availability

The original contributions presented in this study are included in the article. Further inquiries can be directed to the corresponding author(s).
